# A Report of Two Cases of Hazards Associated with High Flow Arteriovenous Fistula in ESRD Patients

**DOI:** 10.1155/2018/1686135

**Published:** 2018-04-10

**Authors:** Vipuj Shah, Rakesh Navuluri, Yolanda Becker, Mary Hammes

**Affiliations:** ^1^Department of Medicine, Section of Nephrology, University of Chicago, Chicago, IL, USA; ^2^Department of Interventional Radiology, University of Chicago, Chicago, IL, USA; ^3^Department of Transplant Surgery, University of Chicago, Chicago, IL, USA

## Abstract

High flow arteriovenous fistulas are a common clinical entity affecting patients with end-stage renal failure receiving hemodialysis. Given the difficulty in predicting who will develop a high flow arteriovenous fistula the exact prevalence is unclear. We present two cases of patients with high flow arteriovenous fistula that developed clinical cardiac failure at a time point after the fistula was placed with findings of significant cephalic arch stenosis. Both patients required treatment of cephalic arch stenosis with balloon angioplasty with subsequent surgical aneurism resection. Accurate and timely diagnosis of high flow arteriovenous hemodynamics by prospective monitoring of volumetric flow and cardiac function is required to halt this process prior to cardiac compromise.

## 1. Introduction

The long term outcome of autologous arteriovenous fistula**s** (AVF) remains poor, contributing to patient morbidity and immense economic burden. In a recent large meta-analysis of 12,383 patients with AVF, the primary failure rate was found to be 23%; at one year 40% of patients either had a failed access or required at least one intervention to maintain patency [1]. Guidelines support a peripheral-to-central sequence of AVF construction beginning with a lower arm AVF so as preserve as many sites as possible for future access creation [2]. Unfortunately, the primary failure rate of AVF is most marked in the lower arm [3]. Because of this higher failure rate, many upper arm AVFs are being placed. Upper arm AVF, especially brachiocephalic fistulas (BCF), have a number of long term complications, including higher flow rates and greater incidence of cephalic arch stenosis (CAS), compared with lower arm AVF [4, 5]. A high flow AVF is defined when the volumetric flow is greater than 1,500 mL/min [6]. We review two cases of high flow AVF with complications of CAS, cardiac failure and aneurysms. The discussion that follows reviews important clinical considerations and treatment options for high flow AVF.

## 2. Case Reports

**Figure 1:**
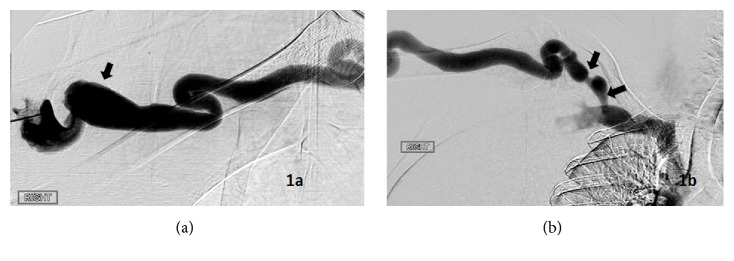
Venogram for Case 1 of venous outflow with tortuosity and aneurysms (arrow) (a). Venogram for Case 1 of cephalic arch with marked tortuosity and tight short segment stenosis (arrows) (b).

*Case 1*. A 36-year-old African American female, with PMH of systemic lupus erythematosus, hypertension, seizures, hypothyroidism, and end-stage renal disease (ESRD) had been receiving hemodialysis for 5 years as a result of end-stage disease secondary to lupus nephropathy. Hemodialysis was started in 2012 via a right IJ permcath with right brachiocephalic (BCF) creation later that same year. The patient received hemodialysis three days a week and began developing access complications in 2015. She was referred to vascular surgery on two occasions because of prolonged bleeding postdialysis. Venograms performed on both occasions showed marked tortuosity of the venous outflow with focal CAS that was treated with angioplasty using a standard 7 mm balloon. In February 2016 she was admitted from the pulmonary hypertension clinic with hypervolemia in the setting of volume overload. Her chief complaint at admission was fatigue and orthopnea, causing her to sleep sitting in a chair for the past two days, and edema. The patient had been unable to obtain her dry weight at dialysis due to hypotension and cramping. In addition she had been having prolonged bleeding postdialysis from her AVF. The patient had been adherent to a dialysis treatment schedule of three days a week and complex medication regime which included amlodipine, clonidine, hydralazine, lasix, labetalol, levetiracetam, levothyroxine, simvastatin, topiramate, and renvela. On physical exam vitals showed a blood pressure of 159/94 with a pulse of 83. Cardiac exam showed normal rate and rhythm, 4/6 systolic ejection murmur heard best at the RUSB with radiation to the carotids which increased with inspiration, and a loud S2. Jugular venous distension was present to the mid neck as was hepatojugular reflex to the anterior ear. Lungs were clear; however 2+ lower leg edema was present. Transthoracic echo showed elevated right ventricular systolic pressure consistent with pulmonary hypertension. The patient was diagnosed with heart failure with preserved ejection fraction (HFpEF) due to diastolic dysfunction and high flow state. The patient was treated with aggressive ultrafiltration with hemodialysis to establish lower dry weight. In March 2016, the patient was again referred to access surgery for prolonged bleeding, difficulty with cannulation and expansile aneurysms. An AVF venous Duplex ultrasound showed volumetric flow measured mid upper arm to be 5180 ml/min. Venogram done at this time showed large aneurysms and tortuous venous outflow (Figure 1(a)) with multiple short tight stenoses in the cephalic arch (Figure 1(b)). This outflow narrowing at the cephalic arch was presumed to be the causative factor for the presenting history of prolonged bleeding and the decision was made to pursue angioplasty. Initial angioplasty was performed using a Mustang 8 mm × 40 mm standard pressure balloon catheter (Boston Scientific, Marlborough, MA) rated up to 20 atm. Postangioplasty imaging demonstrated only moderate improvement. Thus a second angioplasty was performed using a Conquest 8 mm × 40 mm high-pressure angioplasty balloon catheter (C.R. Bard, Murray Hill, NJ) rated up to 40 atm. The more peripheral stenosis opened to 5.5 mm from 4 mm, which equated to a 27% improvement in stenosis. The more central stenosis opened to 5 mm diameter from 2 mm and equated to a 60% improvement in stenosis. Postangioplasty venography demonstrated subjectively improved flow and satisfactory resolution of the stenoses. Due to the high flow state and aneurysms the patient was taken to the OR in April 2016 and the two aneurysms were resected and brought together with end to side anastomosis. Over the following months, the patient returned for angioplasty of the cephalic arch stenosis on two occasions but had marked improvement in symptoms of heart failure, with follow-up venous Duplex ultrasound on Jan 30, 2017, which showed volumetric flow measured mid upper arm to be 1244 ml/min.

**Figure 2:**
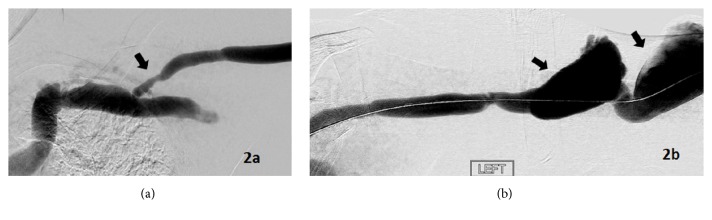
Venogram for Case 2 of cephalic arch with long segment stenosis (arrow) (a). Venogram for Case 2 of venous outflow with marked aneurysms (arrows) (b).

*Case 2*. A 53-year-old African American male with PMH of hypertension and drug use had been receiving hemodialysis for 6 years as a consequence of end-stage renal disease secondary to hypertension. After failed attempts at a left lower nondominate arm AVF, a left BCF was placed in 2011. Over the years the BCF developed large aneurysms at sites of repeated cannulation. In 2015, the patient presented to the Emergency Room with chief complaint of altered mental status. Symptoms at presentation included disorientation to place and time and missed medication and inability to care for himself. He had been receiving hemodialysis three days a week. Clinically during the preceding weeks, the patient was experiencing marked dyspnea with exertion and lower extremity edema, cramping with hemodialysis and inability to achieve prescribed dry weight. Medications on admission included amlodipine, sensipar, hydralazine, megace, pantoprazole, thiamine, and renvela. Physical exam showed a blood pressure of 198/110 a pulse of 106. He was oriented only to person. Lungs were clear, cardiac exam normal S1, S2, 2+ edema. Left AVF was very dilated from the anticubital area to the mid arm. There were multiple large aneurisms which were nontender, thinning skin, but there is no evidence of excoriation. The workup including CT was negative for an intracerebral event. Blood cultures were drawn and came back positive with growth of alpha hemolytic strep treated with intravenous vancomycin and cefepime for 6 weeks. The source of the bacteremia was unclear and a venous Duplex of the access and an echo was done. The AVF Duplex ultrasound study showed a volumetric flow of 10,731 mL/min measured in the mid venous outflow. An echocardiogram done showed moderate LVH, moderate dilation of the right atrium, and marked increase in left atrial volume. Patient was discharged and at subsequent hemodialysis the patient was found to have elevated venous pressures which prompted interrogation via a fistulogram. The venogram study was done in June 2016 with findings of long segment tight CAS (Figure 2(a)) and two large aneurysms (Figure 2(b)). The outflow narrowing at the cephalic arch was presumed to be the causative factor for the presenting history of elevated venous pressures at dialysis and the decision was made to pursue angioplasty. Initial angioplasty was performed using a Mustang 7 mm × 40 mm standard pressure balloon catheter (Boston Scientific, Marlborough, MA) rated up to 20 atm. Postangioplasty imaging demonstrated only moderate improvement. Thus a second angioplasty was performed using a larger Mustang 9 mm × 40 mm standard pressure angioplasty balloon catheter (Boston Scientific, Marlborough, MA) rated up to 18 atm. The cephalic arch stenosis opened up to 6 mm from 3.5 mm, which equated to a 42% improvement in stenosis. Postangioplasty venography demonstrated subjectively improved flow and satisfactory resolution of the stenosis. In August 2016 the patient underwent revision of the left upper extremity AVF with a jump graft inserted, followed by interval ligation and excision of two large pseudo aneurysms. After surgery, the AVF was able to be used with hemodialysis delivered at a 450 mL/min blood flow without the need for central venous catheter access. The patient's volume status was better managed after the AVF revision and subsequently went on to receive a renal transplant in May 2017.

## 3. Discussion

The two cases reported summarize characteristics and complex clinical complications of heart failure, aneurisms, and CAS. These three problems occur commonly together and while a cause and effect are not clearly defined, we are concerned that the high flows diagnosed in upper arm AVF contribute to pulmonary hypertension and high output heart failure and ultimately contribute to CAS. Cephalic arch stenosis once it develops is very difficult to treat often requires repeat angioplasty and stents and may lead to access failure [5].

The diagnosis of a high flow AVF is complicated [7]. One needs to have a high index of suspicion and then determine the volumetric flow in the AVF and the cardiac output. When determining the volumetric flow of the AVF, it is commonly measured in the proximal, mid, and distal venous outflow which may differ dramatically. The volumetric flow should be measured at the feeding brachial artery as it is most reproducible, there is ease of brachial artery imaging and there is less turbulent blood flow when compared to Doppler flow measured throughout the venous conduit [8]. Ko et al. have devised a “Fast, 5-min Dialysis Duplex Scan” which correlates with brachial artery volume flow as a predictor of dialysis access flow maturation and successful hemodialysis [8]. While we acknowledge the limitation of not having brachial artery volume flow available for our case reports, we recommend that the brachial artery be used in future Duplex studies when evaluating an AVF for volumetric flow. High output heart failure may be diagnosed with a transthoracic echo but may require a right heart catheterization for definitive diagnosis [9]. A simple clinical maneuver that can easily aide in the diagnosis is the Nicoladoni-Branham sign [10]. This is performed by occluding the AVF for 30 seconds which may cause bradycardia and increased blood pressure which occurs as a result of the normalization of the cardiac output with AVF compression. Nonetheless, the diagnosis must be confirmed as if high output heart failure is left untreated; it will contribute to cardiovascular and access complications.

High flow within a vascular access for hemodialysis develops over time. As an AVF ages, increased flow within the artery and vein induces dilation, resulting in a gradual reduction in resistance. The resultant high flow circuit leads to altered hemodynamics causing intimal hyperplasia. Intimal hyperplasia results in venous stenosis which causes increased venous pressure, aneurysms, eventual decreased blood flow rate, and venous thrombosis. This, in turn, results in the need for a multitude of access procedures to maintain patency and ultimately may result in access failure. High flow in an AVF will lead to pathological accelerated access growth and cardiac overload. In the BCF all blood flow is committed to traversing the cephalic arch unless there are penetrating veins that communicate with the basilica system. Flows as great as 2,000 mL/min may enter the axillary vein and high flow of over 1000 mL/min predict CAS [11, 12]. BCF access have an increase in cross-sectional area and a dramatic increase in blood flow (mean 1,983 ± 1,199 ml/min) when compared to lower arm AVF [13]. The cases presented illustrate the detrimental sequelae of high flow in an upper arm BCF.

Previous studies have shown that wall shear stress (WSS) plays a significant role in regulating the function of endothelial cells [14]. High and laminar WSS is related to normal endothelial function which has anti-inflammatory and antineointimal hyperplasia activity [15]. With high flow, there are low flow pockets that occur in the curves of vessels as evident in the cephalic arch [16]. Low flow causes disturbed WSS which is associated with vascular endothelial dysfunction resulting in neointimal hyperplasia and a predisposition to the development of CAS [17]. Occurrence of neointimal hyperplasia has an inverse relationship with WSS and is also related to flow patterns [18]. The physiology proposed is that, after arteriovenous fistula creation, high flows cause increased pressure and wall shear stress necessary for AVF maturation. With time low shear stress, described as recirculation zones, develops in the curve of the arch which causes endothelial cell dysfunction and resultant cephalic arch stenosis [16]. As the venous stenosis impedes flow, pressures rise. CAS creates a vicious cycle of high pressure causing tortuous veins and aneurysms. Over time, these extremes cause an abnormal complex interplay between biologic factors that induce outward remodeling and physical factors of wall tension with the end result of the mega-fistula [7].

Cardiovascular disease remains the number one cause of death in ESRD patients. Both traditional risk factors such as diabetes, peripheral vascular disease, and hypertension and nontraditional risk factors such as increased inflammatory markers, homocysteine, anemia, hyperparathyroidism, endothelial dysfunction, and abnormal lipoprotein B are commonly found in hemodialysis patients and can increase cardiovascular mortality [19]. Studies have shown that a 15% increase in cardiac output occurs early after AVF creation by the seventh day [20]. There is a relationship between cardiac output and high flow in an AVF leading to late complications such as high output heart failure manifest as increased left ventricular end-diastolic volume and pulmonary hypertension [21–26].

According to K/DOQI guidelines, asymptomatic aneurysms do not require intervention and should be managed by avoiding cannulation of the aneurysmal areas [2]. Careful cannulation techniques using rope and ladder or area cannulation are best practice when the AVF is mature for use to prevent aneurysms. Management of AVF aneurysms is generally based on clinical signs and symptoms, assessment of overlying skin, ease of cannulation, and functionality of AVF. The diameter of the aneurysm is not an indication for treatment. Venography can define anatomic anatomy of the aneurysm and the presence of upstream venous stenosis such as CAS. The main indication for treatment is based on clinical presentation. Bleeding from an AV aneurysm can be severe and life-threatening. It can occur spontaneously, after hemodialysis treatment when needles are removed or secondary to trauma. Some of the predisposing risk factors to bleeding are thinning or erosion of overlying skin layer, rapidly expanding aneurysm, anticoagulation, prolonged bleeding time after removal of hemodialysis needles, and increased intra-access pressure. In patient with prolonged active bleeding with the above-mentioned signs, immediate surgery is essential. Inadequate dialysis in the setting of aneurysms may be due to as impaired arterial flow or venous outflow stenosis. The treatment is that such cases should be focused on the lesion responsible for low flow and angioplasty would be recommended. However, if there is a risk of bleeding from the aneurysm with associated stenosis, recommended treatments are either an open surgical approach or a covered stent with or without angioplasty. Aneurysmorrhaphy has been an approach to treat high flow AVF resulting in high output congestive heart failure [27]. Multiple surgical treatment techniques have been described in the literature but it is difficult to compare treatment modalities due to lack of randomized control trials.

Any ESRD patient with a history of congestive heart failure or progressive left ventricular hypertrophy should have vascular access flow measured. Access flow may be measurement monthly in the clinical setting using a saline ultrasound dilution method (Transonic Hemodialysis Monitor HDO2 Transonic Systems Incorporated, Ithaca, NY, USA) [28]. We acknowledge the limitation of not providing intradialytic measurements of cardiac output in the present study. The transonic device provides a wealth of longitudinal information that would help to identify and prevent high output AVF and cardiac failure. The ultrasound dilution technique can be used to determine both the access flow (greater than 2 L/min) and the cardiac output (greater than 5 L/min). An access flow to cardiac output ratio greater than 25% may be associated with increased risk of high output cardiac failure [22, 26]. When high access flow is detected and correlated with adverse outcomes such as CAS, high output heart failure, and steal syndrome a difficult decision must be made and every attempt to salvage the AVF. Banding has been shown to be an effective intervention to decrease flow and treat steal syndrome in high flow AVF [29–31].

High flow AVF is a common clinical problem that has been loosely defined, but no doubt contributes to cardiac risk and access complications. Prospective trials to follow the volumetric flow in AVF access and its relationship to cardiac dysfunction are needed. We need to define and then optimize blood flow to prevent these long term complication associated with AVF access.
